# “It doesn’t exist…”: negotiating palliative care from a culturally and linguistically diverse patient and caregiver perspective

**DOI:** 10.1186/s12904-018-0343-z

**Published:** 2018-07-02

**Authors:** Emma Kirby, Zarnie Lwin, Katherine Kenny, Alex Broom, Holi Birman, Phillip Good

**Affiliations:** 1 Practical Justice Initiative, Faculty of Arts and Social Sciences, University of New South Wales, Level 3 Goodsell Building, University of New South Wales, Sydney, NSW 2052 Australia; 2 Royal Brisbane and Women’s Hospital, Brisbane, QLD Australia; 3 University of Queensland, Brisbane, QLD Australia; 4 St Vincent’s Private Hospital Brisbane, Brisbane, QLD Australia; 5 Mater Health Services Brisbane, Brisbane, QLD Australia

**Keywords:** Palliative care, Cultural and linguistic diversity, Qualitative, Australia

## Abstract

**Background:**

The end of life represents a therapeutic context that acutely raises cultural and linguistic specificities, yet there is very little evidence illustrating the importance of such dynamics in shaping choices, trajectories and care practices. Culture and language interplay to offer considerable potential challenges to both patient and provider, with further work needed to explore patient and caregiver perspectives across cultures and linguistic groups, and provider perspectives. The objective of this study was to develop a critical, evidence-based understanding of the experiences of people from Culturally and Linguistically Diverse (CALD) backgrounds, and their caregivers, in a palliative care setting.

**Methods:**

A qualitative study, using semi-structured interviews to explore key experiences and perspectives of CALD patients and caregivers currently undergoing treatment under oncology or palliative care specialists in two Australian hospitals. Interviews were digitally audio recorded and transcribed in full. A thematic analysis was conducted utilising the framework approach.

**Results:**

Sixteen patients and fourteen caregivers from a range of CALD backgrounds participated in semi-structured interviews. The research identified four prevalent themes among participants: (1) Terminology in the transition to palliative care; (2) Communication, culture and pain management; (3) (Not) Talking about death and dying; and, (4) Religious faith as a coping strategy: challenging the terminal diagnosis.

**Conclusions:**

CALD patients and caregivers’ experiences are multifaceted, particularly in negotiating linguistic difficulties, beliefs about treatment, and issues related to death and dying. Greater attention is needed to develop effective communication skills, recognise CALD patients’ particular cultural, linguistic and spiritual values and needs, and acknowledge the unique nature of each doctor-patient interaction.

## Background

The transition to palliative care is challenging for patients and their loved ones [1, 2]. Culturally and linguistically diverse (CALD) populations may need extra support [3], as their experience of transition may entail additional complications around language and cultural values, including barriers to communication and isolation from the broader community. While doctor-patient-caregiver communication around transitions to palliative care has been shown to be complex and prone to interpersonal difficulties [1, 4, 5], cultural and linguistic diversity further complicates doctor-patient communication and treatment options, and the decision to cease potentially life-prolonging treatment [4, 6–9]. In the transition to palliative care, and cultural expectations around death and dying more broadly, CALD patients and families must navigate a range of challenges across the domains of formal and informal care. This study aimed to explore the experiences and perspectives of both CALD patients and family caregivers, revealing various implications for practice for oncologists and palliative care specialists treating CALD patients in the Australian hospital system. Specifically, we focus on the transition to palliative care.^1^ to explore attitudes towards, and understandings and experiences of, end-of-life care among CALD patients and their caregivers.

### Cultural and linguistic diversity when approaching the end of life

Australia is culturally and linguistically diverse, with a range of cultural values and beliefs shaping illness experiences, access to care and interactions with healthcare providers [10]. CALD populations face particular challenges when it comes to accessing healthcare in Australia and in most western nations, including issues related to language and communication, cultural preferences, and isolation [10–12]. In relation to end of life care, patients and caregivers from CALD backgrounds have varying preferences regarding the disclosure of diagnosis and prognosis and the management decision-making in health/care [13, 14]. Internationally, there have been some attempts to understand the particular needs of diverse populations in end-of-life care [15, 16], yet further in-depth, patient-focused work still remains necessary [17, 18].

### Cultural expectations, decision-making and care

Although full disclosure of diagnosis and prognosis is not uniformly practiced in all cultures, it is central to the Western biomedical system [19]. In Australia, doctors are trained in breaking ‘bad news’ to patients and families, particularly when transitioning patients to palliative care [20, 21]. This system is based on the principles of medical ethics, with the notion of ‘patient autonomy’ at its centre [22, 23]. As such, the right of the patient to know their health status and make decisions about treatment options is considered a key facet of medical care. However, some CALD patients and caregivers – particularly those who are older or less ‘acculturated’ to western biomedical contexts – may prioritise a family decision-making model when it comes to treatment decisions [22, 24], or indeed, may defer decisions to their doctors who may be afforded significant cultural authority [7, 25, 26]. As such, Western biomedical notions of patient-centred care may not resonate with patients and caregivers from different cultural backgrounds who have considerably different expectations of medical systems [26–28].

### Communication, culture and palliative care

Doctor-patient communication around diagnosis/prognosis and treatment decision-making is informed by a range of lay and expert interpretations of illness and mortality. Lay interpretations in particular are highly culturally variable and can pose particular challenges for CALD patients and caregivers. For example, the words ‘cancer’ or ‘palliative’ are avoided across a number of cultural contexts because of their direct association with death [25, 29]. There are several explanations for this, including: persistent taboos around cancer and mortality, the idea that speaking of cancer will hasten death, and the idea that the timing of death can be known only by god [25]. Diagnostic/prognostic disclosure is thus a culturally-laden form of communication with considerable implications for palliative care. Furthermore, in the palliative care context, communication necessarily extends beyond doctor-patient relations to include the family, which is often viewed as the appropriate ‘unit of care’. Yet in CALD contexts, family caregivers may be called upon to perform multiple types of caring roles: It is not unusual, for instance, for family caregivers to take on the role of interpreting during medical examinations [30]. Despite the greater accuracy of professional interpreters in conveying information to patients [31], many patients prefer the familiarity of family caregivers [7]. However, the use of family members as interpreters can also be fraught, with tensions around who controls the actual consultation process, and the information conveyed between doctor and patient as mediated by the interpreting caregiver [25, 30, 32]. As we discuss below, such multifarious and complex factors may result in additional confusion, misunderstandings and frustrations in the transition to palliative care for CALD patients and caregivers.

## Methods

This study, while discrete, was part of a broader program of research exploring the lived experience of the end of life. We used qualitative semi-structured interviews to explore the experiences of CALD patients’ and caregivers’ transition to palliative care. Following local and national ethics approval [HREC approval: HREC/14/MHS/22], potential participants were approached by the clinical members of the research team during routine clinic appointments or during inpatient stays at two metropolitan hospitals in south-east Queensland, Australia. When appropriate, potential participants were approached with the aid of an accredited professional interpreter.^2^ Each patient and/or caregiver was given ample opportunity to discuss the study and the context of the interview. Participants who were interested in taking part in the study were then contacted to schedule a convenient time and location for the interview to take place. Eligible participants were people over the age of 18 from CALD communities, defined as those from non-Indigenous ethnic/language groups other than the English-speaking majority in Australia. Patients were approached based on their CALD status as noted in hospital records, who were currently undergoing treatment under oncology or palliative care specialists within the two hospitals. While we did not limit recruitment to specific diagnoses or stage of disease, eligible patients were those who were no longer undergoing potentially curative treatment, and were being transitioned to palliative care. Caregivers were approached based on their provision of care for patients from CALD communities, or based on their own CALD status.

A purposive sampling strategy was used, and coordinated through whole research team meetings, including the clinical members of the research team who approached participants. The process was iterative, and regularly reviewed to update the strategy and facilitate discussion around which patients and carers to approach in order to include participants from a range of backgrounds, including spread across age, sex, languages spoken, inpatient/outpatient status, and public/private care settings. All interviews, conducted during 2014, were digitally audio-recorded and transcribed in full in English.^3^ During the interviews we focused on the following domains: experiences and understandings of palliative care; communication, needs and values in the context of hospital care; experiences of being cared for/caring at a time of serious illness; and, experiences and perspectives on formal/institutional support/care for patients and caregivers with CALD backgrounds. The COREQ qualitative research reporting checklist was used to ensure comprehensive reporting [33].

### Analysis

Data analysis took place concurrently with the qualitative fieldwork over a period of 7 months. In line with sociological interpretive traditions, our approach to analysis focused on reaching a nuanced understanding of the range of perspectives, within systems of beliefs and life experiences more broadly [34]. We employed the framework approach of qualitative data analysis using NVivo 10 software to systematically analyze the transcripts. We employed the following steps: (1) Familiarization: the researchers reviewed the manuscripts. (2) Identification of framework: key themes were identified around which the data were organized. (3) Indexing: application of themes to text. This stage involved using NVivo 10 to label and arrange each excerpt of text, use of a word or term, or research note related to each participant and transcript. This produced several (complicated and overlapping) lists including data and notes from several participants according to each theme. (4) Charting: use of headings and sub-headings to build up a picture of the data as a whole. Each thematic ‘index’ was discussed by three research team members, with Author A leading the development of summaries or ‘charts’ for each thematic area: one ‘overall’ summary, along with one summary of patient participants and one for caregiver participants. (5) Mapping and interpretation: in which associations were clarified and explanations developed. This involved finding associations between and within themes, moving towards and developing explanations for the findings in line with our research aims [35]. The three research team members participated in all stages of the analysis and independently coded the data, and these separate analyses were then cross-checked to uncover related and/or deviant cases/themes, and to develop an overall interpretation of the data. Once a theme was identified in the transcripts, interviews were searched for related comments using constant comparison in order to further develop themes [36]. In this way the richness of the data was retained, while interrelated associations were uncovered. Analytical rigor was augmented by constant comparison and searching for negative cases during code and theme development [35–37]. Following ongoing concurrent analysis of the interviews, informed by the concept of saturation in qualitative research [38]. the researchers agreed that no new themes relating to the topics of study were likely to be identified from further interviews.

## Results

Participants were recruited from 1 public hospital, and 1 private hospital which had provision for public patients through government-funded beds within the private setting. Twenty-two patients and 17 caregivers volunteered to participate, 30 of whom were recruited (16 patients and 14 caregivers^4^). All of the patients had advanced cancer, were no longer receiving potentially curative treatments, 8 of the patients were hospital inpatients at the time of interview, and 8 were outpatients. The caregivers were all family members of the patient for whom they were caring, including daughters (*n* = 4), wives (*n* = 4), husbands (*n* = 4), a granddaughter, and an ex-wife. Several participants spoke multiple languages; the majority of participants spoke some English. Five patients and 5 caregivers were interviewed with the assistance of a professional accredited interpreter. These participants were given a participant information and consent form in a language of their choosing, and were given time to ask any questions about the project and their participation through the interpreter present, prior to giving written consent. Four of the caregiver participants were caring for patients who were also participating in interviews. For these patient-carer dyads, participants were given the option to be interviewed separately or together. Two of patient-carer dyads chose to be interviewed together. Semi-structured interviews lasted up to 60 min (median: 33 min). Table 1 provides a summary of participant characteristics.

**Table 1:** Characteristics of the sample

Characteristic	Patients (*n* = 16)	Caregivers (*n* = 14)
Age		
20–29	–	1
30–39	1	1
40–49	1	4
50–59	3	1
60–69	7	7
70–79	2	–
80–89	2	–
Sex		
Male	5	4
Female	11	10
Religion		
Baptist	–	1
Buddhist	2	1
Catholic	3	4
Christian	2	1
Greek Orthodox	1	1
Hindu	1	–
Jehovah’s Witness	1	–
Latter Day Saints	1	–
Muslim	1	1
None/Not Disclosed	3	5
Pentecostal	1	–
Care type		
Public	12	8
Private	4	4
Not Disclosed	–	2
Regional origin		
Eastern Europe	–	1
European Union	6	2
Oceania	3	1
Asia	5	8
Africa	2	–
Middle East	–	2
Languages spoken		
Afrikaans	1	–
Arabic	–	2
Cantonese	1	2
Dutch	1	–
English	12	11
French	–	1
German	3	–
Greek	1	–
Hindi	1	–
Hungarian	2	–
Italian	2	1
Macedonian	–	1
Mandarin	4	5
Melanesian	1	–
Malay	1	–
Polish	1	–
Somali	1	–
Tagalog	–	1
Taiwanese	1	–

Here we report on the emergent themes following our systematic analysis of the interview transcripts. Participants offered varying degrees of information about their experiences and perspectives on their cancer, supportive and/or palliative care. What was clear, throughout the participant cohort of both patients and caregivers, was that managing hospital treatment for serious illness had the potential to entail significant physical and emotional vulnerability, compounded by communication problems and cultural divergence. Our analysis revealed four predominant themes: (1) Terminology in the transition to palliative care; (2) Communication, culture and pain management; (3) (Not) Talking about death and dying; and, (4) Religious faith as a coping strategy: challenging the terminal diagnosis.

### Terminology in the transition to palliative care

A key theme within the interviews was the variable, or complete lack of, understanding of the concept and meaning of palliative care. Given that there is no equivalent term for ‘palliative care’ in many languages, the majority of participants experienced difficulties in understanding the meaning of the term, and the consequences of the transition to palliative care for treatment and prognosis. As shown in the indicative quotes in Table 2, the expectation of more traditional forms of home-based care for the dying (provided by family or informal caregivers) meant that hospital-based palliative care was difficult to comprehend for several participants. At times, patients confused in-patient palliative care with ongoing ‘active’ treatment that would be aimed at prolonging their lives. That is, some patients believed a return home was indicative of the onset of the dying process and thus, in contrast, that remaining in hospital entailed hope for recovery. Many caregivers lacked a working understanding of the meaning of palliative care, and we heard many accounts of caregivers around ‘doing homework’ in order to understand what ‘palliative’ meant and what ‘palliative care’ entailed. Other participants wished to avoid discussions around terminal prognoses and were reluctant to discuss plans for end of life care. In still other cases (and as with diagnosis), patients and caregivers felt that the meaning of palliative care had been explained by clinicians in ways that were not clear. Thus we found a number of difficulties in participants’ understandings and comprehension of the notion of ‘palliative care’, the precise nature of which varied across patients and caregivers. While various cultural ‘taboos’ around dying played a role, so too did difficulties at the level of translation including the lack of ability to translate and explain the term. What ‘palliative’ meant was, for many participants, lost in translation. Alternatively, in other cases, where ‘palliative’ was taken by participants to be synonymous with approaching the end of life, use of the term was consciously withheld by clinicians at the request of family caregivers.

**Table 2:** Indicative quotations: terminology in the transition to palliative care

Participant	Indicative quotation
Caregiver #1	I’m the type that when you tell me something, especially with regards to my parent’s health … I’ll go and look it up on the internet and research it so that I understand what’s going on so I can help them through it. … even if I had to try and explain it to him using palliative as the word, I couldn’t because it doesn’t exist. It doesn’t exist.
Caregiver #4	*Interviewer: How did the staff explained palliative care to you?* I find it out from the web. Yeah, I find it out from the web. They have explained, but I was still a little bit puzzled by the explanation.
Caregiver #2	*Interviewer: Looking back, do you recall what your understanding of palliative care was before your husband came to [Palliative care unit]?* Special cancer.
Patient #11	Actually, I don’t know. I don’t think I really know what the word palliative means.
Patient #1	P1:They’ve never heard of it [palliative care] … No. I mean they will give you all the medicines that they can afford and if they can’t do anymore then people normally they will just go back to the Island or the village where they come from and just ready to die. They just, just accept it.
Caregiver #3	The thing is, my grandmother, I don’t think that she knows what palliative care means. I think my aunties were trying not [to] tell her that she’s going to die or something… They don’t want her to get upset and stuff like that. She doesn’t really know what it means to be in palliative care. That’s the main thing. She thought that she’s here, I don’t know, maybe she’ll get better, she said.
Caregiver #9 (via interpreter)	Her understanding is that palliative care is for the patients who can’t, at the moment because she can still look after him medicine-wise, can still give him the medications he needs, but because the palliative care is for people out of that, what do you call it, too far gone, I guess. That’s her understanding of palliative care.

### Communication, culture and pain management

A key facet of the transition to palliative care in Australia is the focus on pain and symptom management for patients with advanced cancer [39, 40]. During the interviews with both patients and caregivers, we heard multiple accounts of the difficulties in communicating with clinicians in terms of expectations of treatment, which were shaped by the variable understandings of and meanings attached to palliative care (discussed above). This was most evident in relation to pain and symptom management, with the interviews revealing a variety of different cultural scripts around the expectation of pain and pain relief, as shown in Table 3. As noted above, a significant proportion of the patients we interviewed talked of their belief that by virtue of being in hospital and receiving hospital-based expert medical treatment, they might or would get better and recover. Moreover, the majority of participants (both patients and caregivers) discussed the importance of stoicism and tolerance in the face of physical pain. Thus, participants told us that, when asked about pain by clinicians, patients were likely to down-play the extent of their pain, potentially making it difficult for clinicians to manage pain effectively, or indeed understand the patient experience. One participant provided a poignant anecdote about how this initial hesitation was overcome by a nurse who persisted with their queries until the patient finally felt they could rely on them and fully disclose the extent of their discomfort. For others, communication with clinicians about pain and symptoms was very difficult in the absence of an interpreter. For a few participants, this was experienced as frustrating and isolating, as they felt they could not adequately communicate their needs, nor were their needs well-understood by clinicians. Caregivers discussed similar issues with us, and were often particularly concerned that the Australian medical system was not well equipped to deal with either their cultural or linguistic requirements.

**Table 3:** Indicative quotations: Communication, culture and pain management

Participant	Indicative quotation
Caregiver #1	I’m like their nurse, I’ll go and look it up on the internet and research it so that I understand what’s going on so I can help them through it. I’m the one dealing with the doctors and with medications and all of that.
Caregiver #7	Speaking to that doctor when I went once myself I think there was a communication issue. He sort of said that he didn’t feel that he didn’t get that she was in as much pain as she was ... I think a lot of people from other parts of the world where medical care isn’t so good they seem to have a greater tolerance for pain than people that are used to getting painkillers and whatever. So I think that’s part of the problem in that she didn’t express to him in a way that he comprehended how much pain she was in.
Patient #8	… I never knew you don’t have to pain like you are paining, if that makes sense. I don’t think that’s good English. The pain that I’m going through [prior to pain medication], you don’t have to have that.
Patient #14	When I had this pain in my fingers to start with to get out of that they gave me steroids and morphine... I thought steroids were for horses but they were for me too. In the end I thought, “Why make life more difficult than what you’ve already had? Why not accept?”
Patient #4 (via interpreter)	What has happened she said, when I was new I used to get interpreted but for sometimes now, each time I come they said, “We couldn’t find an interpreter, blah, blah,” a bit of excuse that they make…Yeah, it’s hard. Because I don’t understand what the doctor tells me and the doctor doesn’t understand what I tell him what happened if there’s no interpreter.
Patient #16	Nobody there to listen to you. So then I spoke to doctor himself and I told him. I said, “Doctor this is very painful.” New doctor had come. Now that guy also gave some bloody shitty medicine or something. Then he turns around, 2 days later, he said, “It will take time to go because what I’ve given you is just a painkiller.”

### (Not) talking about death and dying

The avoidance of talking about cancer, death and dying was a predominant theme within the participants’ accounts, and one that was explicitly linked to cultural background (see Table 4 for indicative quotations). Talking about death and dying, or terminal illness was frequently associated by participants with ‘giving up’ or hastening death. That is, participants understood death to be more likely to happen, or more likely to happen quickly, if openly acknowledged, verbalised or discussed. As such, participants spoke of strictly limiting talk about cancer or the end of life, as it was in stark contrast to the strong desire to maintain hope. Both patients and caregivers within our sample spoke of their unwillingness to talk about the prospect of dying as part of their culture or underlying belief system. Such discussions were described as being viewed as demoralising and unnecessarily upsetting for those who are already unwell. Such active avoidance of discussing the terminal diagnosis was also linked to issues around disclosure of information, as well as religion, faith and hope, as we discuss below.

**Table 4:** Indicative quotations: (Not) Talking about death and dying

Participant	Indicative quotation
Caregiver #11	It’s not in our culture to say you’re going to die. I don’t even want to accept myself still. I know that she is ill but I’m still thinking you never know. Miracle may happen or something. It’s hard but I will never tell her. She doesn’t know now. She doesn’t know. I don’t think I would like her to know because she’ll be scared or something. But in our culture you never say to the patient.
Caregiver #1	Sometimes being completely honest and brutal like that is not the right thing to do from a cultural background … I would have told him, “Look dad, there is a tiny little spot that they found there. They’re going to investigate it further. We don’t know exactly what it is. You’re in good hands.” … dad thinks that by talking about death or telling him he’s dying, that you’re going to bring it on, bring on the process a lot quicker.
Caregiver #3	In my culture, they don’t really talk about dying. They’re scared of it…They don’t want to think about my grandma’s going to die. They just deal with it day-by-day. They don’t really plan and they don’t really say, “Oh, yeah, she’s going to die soon.” They don’t want to talk about it.
Patient #16	It’s a cultural thing. But cultural thing a lot of sons they would say, “Yes, yes,” kind of a thing and not get so involved. But my children are very involved and I presume they love me… I would [be] more content to have my sons have it [knowledge of the details of the patients’ prognosis] than to me because, you see, it unnecessarily worries me and it doesn’t solve anything. So I would be happy if my sons know everything about it and they gave me information and that’s okay with me because I trust them implicitly.
Patient #5	So we could [not] say that “I am sick, I am so unlucky, I’m an idiot” Because that will influence the effect on us. So we all say that “I am happy, I’m fabulous, I’m so beautiful.” I have to say like this.
Patient #8	It was on [date] and she actually said, “I think it’s cancer.” I remember how shocked I was that a doctor could throw a word around word like that…So to hear the word, I was really upset. How can you throw a word around like that?

### Religious faith as a coping strategy: Challenging the terminal diagnosis

Many patients – often with the support of their caregivers – talked about the centrality of their faith and religion as a means of coping, as shown in Table 5. Patients and caregivers frequently commented on their preference to focus on religious faith (which they associated with hope) instead of medical futility (which they associated with ‘giving up’ and death). As one caregiver noted about the counselling offered to them and their partner, talking about particular problems only made it more difficult to cope. For some patients, religious faith presented a challenge to their doctor’s expertise and authority, with implication that God knows better, or that God would intervene and assist their healing. One participant admonished herself for saying the word ‘cancer’, explaining this this would anger God, and chose instead to attribute any negative wording about her condition to medical reports by prefacing them with ‘according to the report’. Faith and religion was a particularly prominent coping strategy among our participants. These belief systems were frequently talked about by participants as part of the cultural fabric with which experiences of illness and care were interwoven.

**Table 5:** Indicative quotations: Hope and faith: Challenging the terminal diagnosis

Participant	Indicative quotation
Patient #5	So my bone cancer considered very, very dangerous and I shouldn’t say my mine bone cancer, sorry God. I cannot say that I have cancer he will give me bad feeling … I believe God. God say that everything that come out from your mouth, we always have to say positive about us. I’m not sick, I’m healthy, God is healing me. The more we say about negative thing, the more that negativity will come to us.
Patient #16	I believe in God. There’s a particular God, that I believe in. I have 100% faith that he is not going to let me down, that everything’s going to come through correct.
Patient #8	I’m very positive in my approach to my illness. So I believe God is going to heal me. […] Even if I might be on my deathbed, I will still believe the manifestation of my healing will come. That’s how I prefer to die and pass away… I’m going to not to be a statistic of a carcinoma death, but I will be a miracle.
Patient #3	The doctor can’t tell me how many more years or months I have. So I can only sense it myself, feel it myself, I suppose.
Patient #5	I, myself, believe God will heal me. So when Dr. [Name] say, “Nothing we can do.” I say to myself, “Yeeha, God will show His power. He will heal me.”
Caregiver #14	I’m a spiritual person, as in I believe that there is someone looking out for us. Not so that I go to church. But I have a strong sense of faith and my dad has a strong sense of faith and he is a very positive person and that’s what’s been helping him throughout his whole illness because he thinks he’s just going to get better and he’s very positive. So we do, we have a strong sense of faith.

## Discussion

A significant body of existing research has revealed multiple cultural factors which can shape experiences of discussing and managing death and dying [6, 13]. The findings presented in this paper have provided additional insight into the experiences of CALD patients and their caregivers, and how they manage the challenging nexus of formal care; beliefs about treatment, death and dying; linguistic difficulties; communication around diagnosis, prognosis, pain and symptoms, with clinicians. For clinicians, understanding these complex dynamics is important for effectively communicating, establishing trust and making decisions with both CALD patients and their families. This requires a nuanced negotiation and understanding of both the broader cultural perspective of the patient and the interpersonal relationships within the family unit of care.

Our findings novel data from during the transition to palliative care, which support previous research highlighting the difficulties of communicating with CALD patients about the meaning of palliative care [4, 9], as understandings of the context and services offered by palliative care were incomplete or absent for the majority of participants. The data highlights the importance of clinicians’ ability to identify and remedy misunderstandings about (or lack of understanding of) the meaning and context of palliative care, and what patients and caregivers might expect from this stage in the patient’s care plan. Indeed, our findings point to the significance of ensuring the meaning of language is understood, while maintaining a culturally sensitive disposition (though this is no straightforward task). Moreover, given that several participants described their deliberate avoidance of such discussions, our findings provide new insight into the importance of sensitivity to cultural taboos and scripts around cancer, death and dying.

The findings presented here suggest that such cultural taboos are interpreted in a variety of ways with a variety of consequences. While some (sometimes quite specific) cultural taboos exist in relation to discussing cancer or death, the primary reason given by our participants for such avoidance strategies was the desire to maintain hope and, in the case of patients withholding their status from loved ones, to prevent suffering or concern. In many cultures (including in many western contexts) the maintenance of hope is particularly pertinent approaching the end of life [41], where the transition to palliative care may be seen as giving up hope and provides challenges in terms of denial and acceptance [42]. While the majority of participants recounted avoiding talking about the terminal diagnosis, all of the patients we interviewed were receiving treatment and transitioning to palliative care, suggesting difficulties in communicating the full implications of the transition to palliative care (whether because of difficulties of language, conversational reticence, or avoidance as discussed above). That is not to say that patients or their caregivers were ignorant or uninformed about the details of their diagnosis, but rather that they frequently did not wish to verbalise or discuss them. While such accounts at the end of life are hardly exclusive to CALD patients and caregivers [40–43], our findings point to the complex interplay of culture and care in the every day experience of the transition to palliative care for CALD patients and caregivers.

Successfully engaging in discussions with CALD families around palliative care issues, then, is clearly not as straightforward as simply identifying a particular nationality or cultural background and the associated beliefs and norms, which would amount to a form of cultural stereotyping. Instead, a patient or caregiver’s willingness (or disinclination) to discuss cancer and dying can be recognised as necessarily influenced by a multitude of factors, including individual biography, family relationships, educational attainment and cultural background including, most notably in this study, elements of faith and hope. This highlights some of the challenges palliative care and oncology clinicians may face when working with CALD patients and caregivers, and the importance of approaching each case as unique, with good communication between clinicians and the patient about their particular cultural, linguistic and spiritual values and needs. A number of researchers have argued the importance of clinicians actively exploring case-by-case cultural preferences for how end of life contexts are approached, in particular around patient and caregiver preferences for decision-making and information about their diagnosis and prognosis [8, 22, 26].

Our study has various limitations which are somewhat similar to those faced by clinicians involved in the transition to palliative care. Given the linguistic barriers, the sensitive topic of the interviews, and the conducting of interviews within a busy hospital setting (albeit in a private room), conversations between the researchers and participants were at times stunted, short, and interrupted. Our participants were recruited from two hospitals in one Australian city. As such, our findings may not be indicative of the experiences of CALD patients and caregivers elsewhere. Participants self-selected by volunteering, and were from a wide range of cultural backgrounds, with varying levels of health-literacy in relation to the Australian health system. It is noteworthy to stress again that our findings should not be generalised to all CALD patients and caregivers. Indeed, we reiterate here the almost infinite spectrum of experience for those within CALD communities, and the use of ‘CALD’ to describe a static or homogenous group, or indeed as particular categories (e.g. country of birth), as unhelpful. Finally, we note here the complexities of interviews conducted in English with participants for whom English is not their first language, or through an interpreter, and the potential consequences for data collection, analysis and reporting.^5^ The process of cross language qualitative research has been shown to be complicated and often flawed [44]. While we engaged with issues around competence (using only accredited interpreters) and style (using consecutive interpreting), as well as strategies for building rapport (including making the interpreter ‘visible’ and part of the conversation), we acknowledge the limitations of having an interpreter present, and the limitations in terms of translation of data [44, 45].

## Conclusions

CALD patients and caregivers’ experiences are multifaceted, particularly in negotiating linguistic difficulties, beliefs about treatment, and issues related to death and dying. Greater attention is needed to develop effective communication skills, recognise CALD patients’ particular cultural, linguistic and spiritual values and needs, and acknowledge the unique nature of each doctor-patient interaction. Our findings point to some of the shared barriers and challenges experienced by CALD patients and caregivers in the transition to palliative care which can be broadly attributed to a lack of cultural and linguistic concordance with the Australian hospital system, and the clinicians therein, as well as revealing the difficulties in dealing with sensitive topics such as the end of life. Future research focusing on the meanings of various serious, life-limiting or terminal illnesses is needed across a diverse range of CALD patients and caregivers to better understand CALD experiences of the Australian health system and the ways by which culture and biography inflect illness (and care) experience. Further, to explore what can be learned from CALD communities as well as how to include and engage with CALD populations to continue to improve outcomes.

## Abbreviations

CALD: Culturally and Linguistically Diverse
COREQ: COnsolidated criteria for REporting Qualitative research
HREC: Human Research Ethics Committee

## Footnotes

1 We define the transition to palliative care as the referral and transfer to the care of a palliative care service. This transitional process may entail initial or ongoing consultation with a palliative care specialist within the current clinical setting, and may occur alongside potentially curative treatment, but eventually involves movement to another setting under the care of a palliative care service.

2 Participants were approached using an interpreter in cases where participating clinician researchers determined the need for an interpreter as part of the patients care/consultations. That is, if a patient requested, or was deemed by clinicians to require an interpreter for their hospital appointments, we also used an interpreter to approach them about participation in the study.

3 All interviews were conducted by the same research assistant interviewer, a sociologist with experience in conducting qualitative interviews, who was female and from an Anglo-Australian background. The interviewer was supported by the other members of the research team, including regular debriefing, redevelopment of the interview schedule and strategies/approaches to interviewing people within sensitive or vulnerable contexts, and interviewing through interpreters.

4 Thirty nine potential participants were approached in total, all of whom initially agreed to participate; six dropped out of the study following initial interest and prior to taking part in an interview; three participants were not interviewed due to repeated difficulties in scheduling and availability.

5 For example, several of our codes and subthemes were clear when getting a ‘whole’ sense of an interview transcript, but because of differences in linguistic style or proficiency in English among our participants, do not lend themselves straightforwardly to being depicted through quotations within a paper in a conventional sense. A consequence of this is that some participants’ voices are not privileged (or seemingly valued) within this paper, and as a research team we acknowledge the contribution made by all participants, and the value of every participant’s data within our analytic process.
